# Phylogenomics and Spatiotemporal Dynamics of Bovine Leukemia Virus Focusing on Asian Native Cattle: Insights Into the Early Origin and Global Dissemination

**DOI:** 10.3389/fmicb.2022.917324

**Published:** 2022-06-24

**Authors:** Kohei Nishikaku, Takahiro Yonezawa, Masahide Nishibori, Masashi Harada, Fuki Kawaguchi, Shinji Sasazaki, Yasushi Torii, Kazuhiko Imakawa, Kuniko Kawai, Jianquan Liu, Hideyuki Mannen, Tomoko Kobayashi

**Affiliations:** ^1^Laboratory of Animal Health, Department of Animal Science, Faculty of Agriculture, Tokyo University of Agriculture, Atsugi, Japan; ^2^Laboratory of Animal Genetics, Department of Animal Science, Faculty of Agriculture, Tokyo University of Agriculture, Atsugi, Japan; ^3^Laboratory of Animal Genetics, Graduate School of Integrated Sciences for Life, Hiroshima University, Higashi-Hiroshima, Japan; ^4^Laboratory Animal Center, Osaka City University Graduate School of Medicine, Osaka, Japan; ^5^Laboratory of Animal Breeding and Genetics, Graduate School of Agricultural Science, Kobe University, Kobe, Japan; ^6^Laboratory of Molecular Reproduction, Research Institute of Agriculture, Tokai University, Kumamoto, Japan; ^7^Department of Biology, School of Biological Science, Tokai University, Sapporo, Japan; ^8^Key Laboratory for Bio-Resource and Eco-Environment of Ministry and Education, College of Life Sciences, Sichuan University, Chengdu, China

**Keywords:** bovine leukemia virus (BLV), deltaretrovirus, endogenous retrovirus (ERV), virus evolution, retrovirus, Phylodynamics

## Abstract

Bovine leukemia virus (BLV), the causative agent of enzootic bovine leukosis, is currently one of the most important pathogens affecting the cattle industry worldwide. Determining where and in which host it originated, and how it dispersed across continents will provide valuable insights into its historical emergence as the cattle pathogen. Various species in the *Bos* genus were domesticated in Asia, where they also diversified. As native cattle (taurine cattle, zebu cattle, yak, and water buffalo) are indigenous and adapted to local environments, we hypothesized that Asian native cattle could have harbored BLV and, therefore, that they were important for virus emergence, maintenance, and spread. In this study, phylogeographic and ancestral trait analyses—including sequences obtained from Asian native cattle—were used to reconstruct the evolutionary history of BLV. It was shown that, since its probable emergence in Asia, the virus spread to South America and Europe via international trade of live cattle. It was inferred that zebu cattle were the hosts for the early origin of BLV, while taurine cattle played the significant role in the transmission worldwide. In addition, the results of positive selection analysis indicate that yak had a substantially minor role in the transmission of this virus. In this study, endogenous deltaretrovirus sequences in bats, collected in Asian countries, were also analyzed on whether these sequences were present in the bat genome. Endogenous deltaretrovirus sequences were detected from bat species endemic to specific regions and geographically isolated for a long time. Endogenous deltaretrovirus sequences from these geographically isolated species represent ancient exogenous deltaretroviruses distributions. The phylogenetic analysis revealed that these newly obtained endogenous deltaretrovirus sequences were closely related to those of BLV from Asian native cattle, indicating that BLV-related ancient deltaretroviruses circulated in Asia long before the emergence of BLV. Together, our analyses provide evidence for origin and spatiotemporal dynamics of BLV.

## Introduction

The rapid growth of the global cattle industry has been associated with changes in the spatial dynamics and types of pathogens in cattle population. Over the years, the domestication of wild cattle and/or the cross-breeding of indigenous animals with domesticated ones, which affect disease dynamics, resulting in emergence of pathogens. Moreover, the increasing global trade of live animals and selective breeding also elevate the risk of dissemination of pathogenic viruses (Perry et al., 2013).

Bovine leukemia virus (BLV) is one of the emerging pathogens in cattle population, and it belongs to the *deltaretrovirus* genus, *Retroviridae* family (Aida et al., 2013). BLV causes enzootic bovine leukosis (EBL), which is the most common neoplastic disease with B-cell lymphosarcoma in cattle. Although only a small fraction (3∼5%) of BLV-infected cattle develop lymphosarcoma, EBL is responsible for significant economic losses in dairy and beef cattle farms worldwide. EBL is listed as a disease of importance to international trade by OIE (the World Organization for Animal Health) (Ott et al., 2003; Benitez et al., 2020).

EBL in cattle was first reported in Germany in 1878 (Boeke and Stoye, 1997). The disease gradually spread from the initial endemic area of Memel in East Prussia (now Klaipeda, in Lithuania) along the coasts of the Baltic Sea by approximately 1920 (Sihvonen, 2015). During the two world wars, massive international trades of cattle probably contributed to the spread of BLV across Europe and other countries (Bendixen, 1963). In the mid-twentieth century, EBL had been reported in most cattle raising countries. BLV infections in Western Europe have been eradicated and remain free of infection to this day through continuous surveillance and eradication programs (OIE, 2021). However, BLV is still common in Canada, the United States, and many countries in eastern Europe, South America, and Asia (Polat et al., 2017). Previous detailed phylogenetic studies have indicated that BLV can be divided into at least 10 genotypes associated with different geographic distributions (Polat et al., 2017). For example, genotype 1 is found all over the world, genotype 4 is mainly found in Europe and Russia, and genotypes 6 and 10 are mainly found in Asia (Polat et al., 2017). As cell-free viruses are unstable, close physical contact and exchange of infected lymphocytes, or body fluids, are required for BLV transmission (Constable et al., 2017). Therefore, the worldwide distribution of BLV genotypes between distant geographic locations has been probably driven by the virus spreading through the transport of live infected animals.

Given the wide geographic distribution of BLV, little is known about the place or region where BLV originated. Europe is considered as the historical origin of EBL, however, while taurine cattle (*Bos taurus*) was introduced to Europe during the Neolithic period (8,000 years ago), the earliest record of the symptoms associated with BLV dates back to only 140 years ago (Scheu et al., 2015). This historical context raises the possibility that the emergence and rapid spread of BLV in taurine cattle populations occurred recently. BLV predominantly infects taurine cattle, but a number of studies reported that it also infects Asian native cattle, such as zebu (*B. indicus*), yak (*B. grunniens*) and, sporadically, water buffalo (*Bubalus bubalis*) (Ma et al., 2016; Wang et al., 2018). However, BLV dissemination routes relative to roles each host species played remain unknown.

Most agree with the thoughts that bats are reservoirs of emerging viruses. It was shown that bats could harbor exogenous retroviruses (Hayward et al., 2020). In fact, bats have been reservoirs for emerging viral diseases, including Hendra virus, Nipah virus, Marburg virus and Ebola virus, severe acute respiratory syndrome (SARS) virus as well as the current pandemic SARS-CoV-2 (Irving et al., 2021). Therefore, bats could be considered as the comparative phylogeography of widespread, co-distributed species, providing unique insights into regional biodiversity and diversification patterns (Roberts, 2006). Previous studies have reported that endogenous deltaretrovirus sequences are present in the bat genome (Farkasova et al., 2017; Hron et al., 2019). Although bat species are distributed worldwide, some species are endemic to specific regions and geographically isolated for a long time (Millien-Parra and Jaeger, 1999). Endogenous deltaretrovirus sequences from these geographically isolated species represent ancient exogenous deltaretroviruses distributions.

The aim of this study was to gain the information on the area, original host, and dispersal route of BLV, focusing in particular on Asian native cattle. In the present study, BLV infections were epidemiologically analyzed, and BLV proviral sequences were obtained from extensively collected samples of Asian native cattle. Bayesian phylogeographic techniques were used to infer the origin and dispersal routes of BLV. We then combined geographic distribution of bat species and phylogenetic relationships of bat endogenous deltaretrovirus sequences with BLV to further validate Asian origin of BLV. The data generated through this study provide evidence that BLV was transmitted from Asian zebu cattle to taurine cattle and was then disseminated throughout the world.

## Results

### Detection of Proviral Genome and Analysis of the Bovine Leukemia Virus Genetic Lineage in Native Cattle

To analyze the epidemiological distribution and obtain the BLV sequences from Asian native cattle, archived field samples from 256 zebu cattle (*B. indicus*), 16 native taurine cattle (*B. taurus*), 268 yaks (*B. grunniens*), and eight water buffaloes (*Bubalus bubalis*) collected in 11 Asian countries were used. In addition, samples from 37 zebu cattle in Madagascar were also analyzed. Among these samples, the BLV proviral DNA was detected in zebu cattle samples obtained from seven countries (Bhutan, Cambodia, Laos, Myanmar, Philippine, Vietnam, and Madagascar), native taurine cattle samples from Republic of Kazakhstan, and yak samples from three countries (China, Nepal, and Pakistan), while the proviral DNA was not detected in water buffalo samples (Table 1). These results indicate that BLV is widely distributed in Asian native cattle population (within the *Bos* genus).

**TABLE 1:** Detection of bovine leukemia virus (BLV) proviral genome in Asian native cattle.

Species	Countries	Collection year	Samples tested	*n^a^*	*env* PCR positive	*LTR* PCR positive	Positive (%)	(/All)	Obtained sequences	Genotype		
										1	4	10
**Zebu cattle (*Bos indicus*) (*n* = 293)**												
	Bhutan	2010	Blood	47	3	NT*^b^*	6.4	(3/47)	1	1	–*^c^*	–
	Cambodia	2001–2003	Blood	20	1	NT	5	(1/20)	0	–	–	–
	Laos	2011	Blood	47	10	NT	21.3	(10/47)	2	–	–	2
	Myanmar	2001, 2002	Blood	65	8	NT	12.3	(8/65)	3	–	–	3
	Philippines	2019	Blood	29	1	NT	3.4	(1/29)	0	–	–	–
	Vietnam	1996, 1997	Blood	48	3	NT	6.3	(3/48)	1	1	–	–
	Madagascar	2016, 2017	Blood	37	3	NT	8.1	(3/37)	1	–	–	1
**Taurine cattle (*Bos taurus*) (*n* = 16)**												
	Republic of Kazakhstan	2015	Blood	16	7	NT	43.7	(7/16)	5	-	5	-
**Yak (*Bos grunniens*) (*n* = 268)**												
	China	2011, 2013, 2015, 2017, 2018	Skin	97	NT	20	20.6	(20/97)	0	–	–	–
	Kyrgyzstan	2019	Blood	12	0	0	0	(0/12)	0	–	–	–
	Nepal	2018	Blood	72	NT	13	18	(13/72)	0	–	–	–
	Pakistan	2018	Skin	87	NT	16	18.3	(16/87)	0	–	–	–
**Water Buffalo (*Bubalus bubalis*) (*n* = 8)**												
	China	2018	Skin	8	NT	0	0	(0/8)	0	–	–	–
**Total**				585	36	49	14.5	(85/585)	13	2	5	6

*^a^Number of samples tested for BLVgp51 or LTR proviral DNA.*

*^b^Not tested.*

*^c^Not detected.*

**FIGURE 1:**
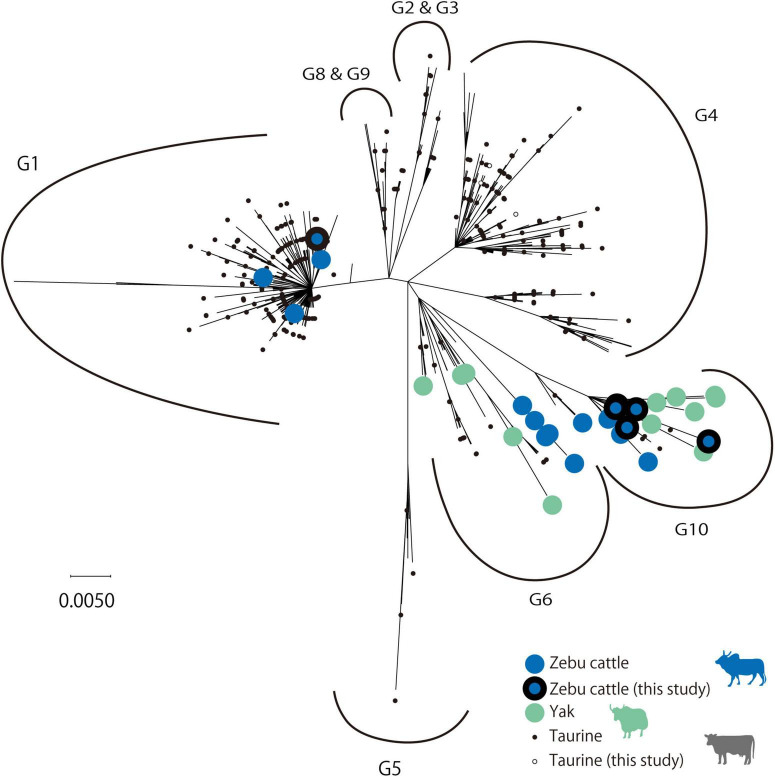
Maximum likelihood (ML) phylogenetic tree of *BLVgp51* partial sequences. ML phylogenetic tree of the *BLVgp51* partial sequences (444 bp) derived from 699 bovine leukemia virus (BLV) sequences. The tree was constructed using the *BLVgp51* sequences from 13 native cattle in this study and 686 sequences from known BLV. The year and the location of sequence data collected were obtained from the GenBank database. Genotypes 1 through 10 are indicated by the letter “G.” Native cattle are indicated by large circles, with zebu cattle and yak being identified by blue and gray colors, respectively; the sequences obtained in this study are surrounded by a black border. Taurine cattle are indicated by a small circle, and the sequences obtained in this study are represented by a white circle.

Among these BLV-positive samples, sequences of the BLV *envelope glycoprotein gp51* gene (*BLVgp51*) were obtained from zebu cattle from Laos (*n* = 2), Myanmar (*n* = 3), Vietnam, Bhutan (*n* = 1), Madagascar (*n* = 1), and native taurine cattle from Kazakhstan (*n* = 5). The maximum likelihood (ML) phylogenetic analysis revealed that all sequences were classified into genotypes that have been previously reported (genotypes 1, 4, and 10) (Figure 1 and Table 1).

### Phylodynamic Analysis of Bovine Leukemia Virus From Asian Native Cattle

**FIGURE 2:**
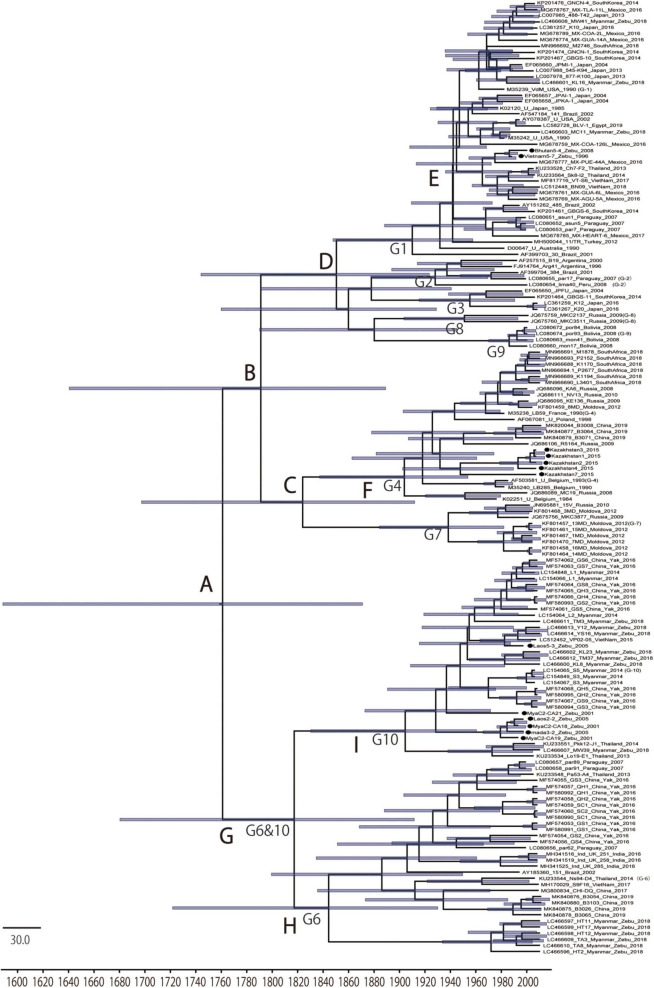
Maximum clade credibility (MCC) tree of *BLVgp51.* Time-scaled maximum clade credibility (MCC) tree inferred from *BLVgp51* sequences (502–903 bp) of native cattle (*n* = 13, indicated by the black circle) and worldwide bovine leukemia virus (BLV) sequences (*n* = 143). Bars at nodes indicate a 95% HPD of tMRCA. The node abbreviations “G1–G10” and “A–I” indicate the BLV genotypes and the node ID, respectively, based on the coalescent event (node ID; Table 2). The sequences obtained in this study are marked by a black circle.

To gain insights into the temporal and spatial dissemination of BLV in Asian native cattle, the full length *BLVgp51* sequences were analyzed using a Bayesian Markov Chain Monte Carlo (MCMC) phylogeographic approach (Figure 2 and Table 2). The maximum clade credibility (MCC) tree revealed that BLV sequences were segregated into two reciprocally monophyletic clusters, lineage I and lineage II, in 1791 (95% HPD: 1640–1889) and 1817 (95% HPD: 1680–1912), respectively (posterior probability = 1). BLV sequences from most zebu cattle and all yak samples obtained in Asian countries fell into lineage II, while sequences from taurine cattle samples from all over the world fell into lineage I.

**TABLE 2:** Time to most recent common ancestor (tMRCA) inferred from Bayesian analysis of BLVgp51 sequences.

Coalescent event	Node ID	Median	(95% HPD*^a^*)
BLV root	A	1761	(1588–1871)
Lineage 1	B	1791	(1640–1889)
Genotype 4 and 7 root	C	1824	(1697–1912)
Genotype 1, 2, 3, 8, and 9 root	D	1850	(1744–1923)
Genotype 1 (United States/Mexico) root	E	1942	(1913–1972)
Genotype 4 root	F	1904	(1837–1954)
Lineage 2	G	1817	(1680–1912)
Genotype 6 root	H	1844	(1830–1961)
Genotype 10 root	I	1904	(1722–1930)

*^a^95% highest probability densities estimates.*

**FIGURE 3:**
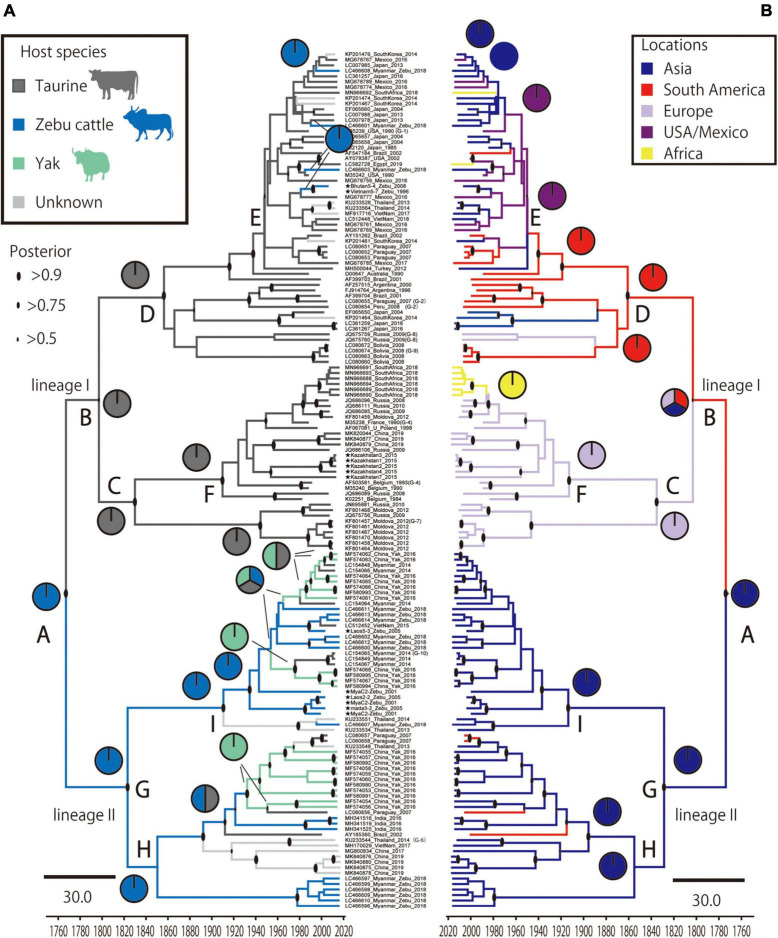
Time scale phylogenetic tree and ancestral state reconstruction. Ancestral states of hosts **(A)** and locations **(B)** in bovine leukemia virus (BLV) were estimated using the maximum parsimony method in Mesquite using the MCC tree. On the left, the node pie-charts and branch colors for the host species indicate taurine cattle, zebu cattle, and yak, respectively; on the right, the node pie-charts and branch colors for the locations show Asia, South America, Europe, United States/Mexico, and Africa, respectively. The posterior probability of the MCC tree is indicated by the size of the black circle at each node. The node abbreviations **(A–I)** indicate the node ID based on the coalescent event (node ID; Table 2).

To estimate where and which host BLV originated, ancestral traits at all internal tree nodes were reconstructed by a parsimony-based algorithm. The results of the analysis using sampling locations as terminal taxa showed that BLV sequences obtained from Asia reconstructed at the BLV root in 100% (node ID: A in Figure 3B), suggesting that BLV originated in Asia. Similarly, the results of the analysis using host species as terminal taxa showed that zebu cattle reconstructed at the BLV root in 100% (node ID: A in Figure 3A), suggesting that the ancestral host of BLV is zebu cattle. Overall, these results indicate that BLV was likely to have originated in Asian zebu cattle.

### Positive Selection Analysis of Bovine Leukemia Virus Sequences in Each Lineage

**FIGURE 4:**
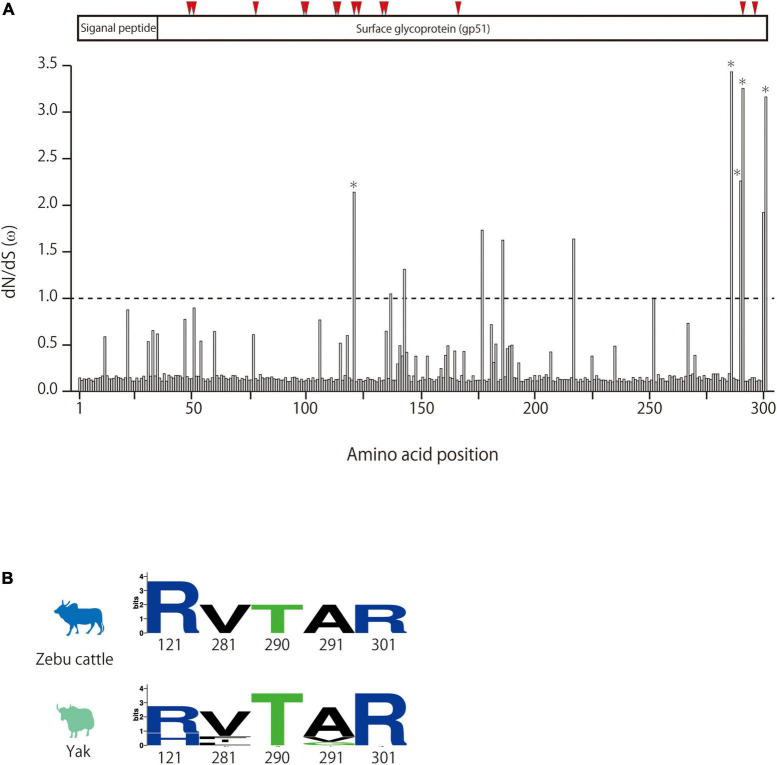
Detection of positive selection pressure of BLVgp51 in bovine leukemia virus (BLV) lineage II using the PAML package site model. **(A)** dN/dS (ω) of BLVgp51 in BLV lineage II analyzed by site model. The Bayes empirical Bayes (BEB) method in the site model were used to infer the amino acid sites under positive selection. Weak evidence of positive selection was observed at five amino acid sites (indicate by *) in BLV lineage II. The proteins coded by BLVgp51 is displayed at the top of the figure. The binding sites for boAP3D1—one of the receptors for BLV—are indicated by red triangles (positions: 48, 50, 77, 98, 99, 112, 120, 135, 136, 187, 292, and 298). **(B)** The amino acid composition of the positive selection of zebu cattle and yak in BLV lineage II is visualized in Weblogo.

To investigate whether lineages I and II appeared because of viral adaptation, BLV sequences from each lineage were separately analyzed for positive selection by using the CODEML site test in the Phylogenetic Analysis by Maximum Likelihood (PAML) program package. The non-synonymous (dN) and synonymous (dS) substitution rates of BLV envelope sequences were calculated. The Bayes empirical Bayes calculation of posterior probabilities in PAML identified five amino acids (121, 281, 290, 291, and 301) as being under positive selection with posterior probability >0.50 in the BLV sequences of lineage II (Figure 4A). The selected sites likely correspond to predicted BLV receptor binding sites (Figure 4A). Among the sequences classified in linage II, variants in three positively selected sites were only detected in BLV sequences from yaks (Figure 4B). No evidence of selection was detected for lineage I (data not shown).

### Identification of Major Bovine Leukemia Virus Migratory Routes

**FIGURE 5:**
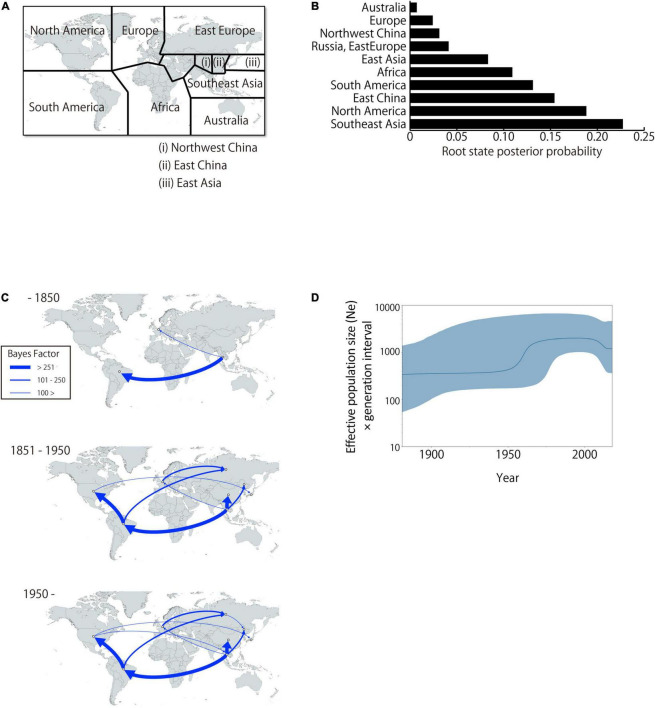
Global dissemination routes and effective population size of bovine leukemia virus (BLV). **(A)** Area delimitation in biogeographic analysis. **(B)** The posterior probabilities for the root state locations. **(C)** Bayesian stochastic search variable selection was used to infer dispersal routes with the Bayes factor. Dispersal routes that presented a significant support (Bayes factor ≥ 3) are plotted. **(D)** Plot depicting changes in the Effective population size (Ne) × generation interval. The dark line shows the effective population size estimated through time. The blue shaded areas correspond to the 95% HPD.

To analyze BLV migration pathways in detail, the sampling locations were divided into 11 geographic areas (Figure 5A). In the results of the root state posterior probabilities (RSPP) of the tree location files obtained from BEAST analysis, Southeast Asia showed the highest RSPP value, indicating that the BLV progenitor is likely to have emerged in Asia (PSPP = 0.277, Figure 5B). Using the discrete phylogeographic model, the BLV dataset analysis identified a total of 46 well-supported transmission routes through the Bayes factor (BF) test using a cutoff value of three (Table 3). Based on the results of the ancestral trait reconstruction (Figure 3B), following the initial emergence of BLV in Asia, the progenitor of BLV lineage I reached Europe in 1824 (95% HDP, 1697–1912, node ID: C), and it subsequently reached South America in 1850 (95% HDP, 1744–1923, node ID: D). Then, the spread of BLV from South America to the United States and Mexico began in 1942 (95% HDP, 1913–1972; node ID: D) (Figure 3 and Table 2). Potential transcontinental BLV spreads were also detected from North America to Japan, from Europe to Russia, and from Southeast Asia to China in 1950 (Table 3 and Figure 3, summarized in Figure 5C). The Bayesian skyline plot analysis indicated that the effective population size significantly increased during the mid-1900s, followed by a slight decline after the 2000s (Figure 5D).

**TABLE 3:** Connections between significant location pairs and their corresponding Bayes factor (BF).

Route No.	BF*	Locations**	
1	1327.3	Northwest China	North America
2	824.1	Northwest China	East China
3	775.7	East Asia	Russia, East Europe
4	635.2	Central Asia	Europe
5	505.8	Central Asia	Northwest China
6	374.2	Northwest China	Southeast Asia
7	373.7	Africa	North America
8	365.0	Northwest China	Russia, East Europe
9	327.4	East Asia	East China
10	309.8	East Asia	Australia
11	306.2	South America	Southeast Asia
12	298.7	Central Asia	East China
13	296.1	Central Asia	South America
14	273.7	South America	North America
15	226.0	East Asia	Northwest China
16	224.1	Northwest China	Europe
17	204.2	Russia, East Europe	North America
18	183.7	Central Asia	Africa
19	181.7	East China	South America
20	181.5	Northwest China	Australia
21	163.8	East China	Southeast Asia
22	160.9	North America	Australia
23	157.5	Central Asia	North America
24	147.2	East China	Africa
25	127.5	Russia, East Europe	South America
26	123.6	Europe	North America
27	120.5	South America	Australia
28	117.3	Northwest China	South America
29	109.7	Central Asia	Australia
30	105.1	Africa	Southeast Asia
31	93.2	Europe	Southeast Asia
32	87.6	Russia, East Europe	Africa
33	75.3	Africa	Australia
34	66.9	Europe	Australia
35	66.7	Russia, East Europe	Europe
36	61.4	East China	Europe
37	58.8	East Asia	Africa
38	46.1	Europe	Africa
39	44.2	East Asia	North America
40	40.6	East China	North America
41	33.3	Central Asia	Russia, East Europe
42	32.4	East China	Russia, East Europe
43	31.8	Northwest China	Africa
44	26.6	East China	Australia
45	26.2	Central Asia	Southeast Asia
46	11.3	Southeast Asia	North America

*Only transitions with BF > 3 are presented.

**Countries or provinces included in each region: East China, Heilongjiang province; Northwest China, Gansu province; East Asia, Japan and South Korea; Southeast Asia, Myanmar, Bhutan, Thailand, Laos, and Vietnam; Europe, Belgium, Poland, Germany, and France; East Europe, Moldova, Africa include South Africa, and Egypt; North America, United States and Mexico; South America, Brazil, Paraguay, Argentina, Bolivia, and Peru (Figure 5C).

### Endogenous Deltaretrovirus Sequences From Asian Bats

**FIGURE 6:**
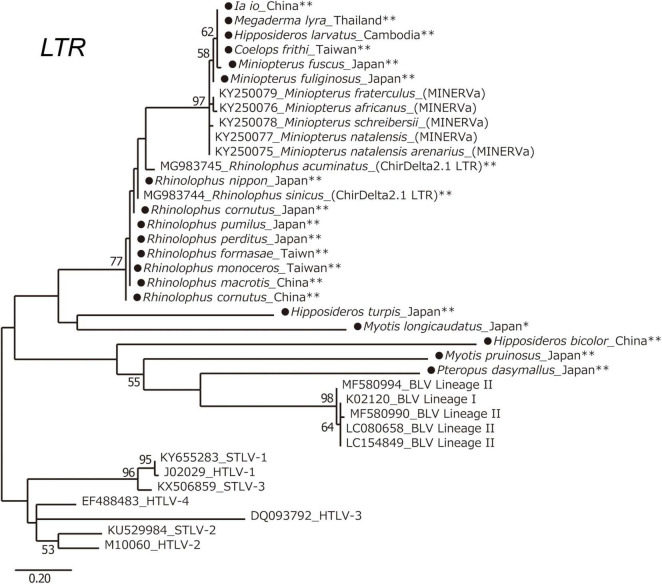
Long terminal repeats (LTR) phylogenetic tree. Maximum likelihood (ML) phylogenetic tree based on deltaretrovirus LTR sequences. A ML phylogenetic tree was constructed from deltaretrovirus LTR sequences derived from 19 bat-specific endogenous deltaretroviruses in this study, and 19 sequences from known exogenous and endogenous deltaretroviruses obtained from GenBank. Sequences were aligned using ClustalW, and the phylogenetic tree was inferred by ML analysis in MEGA7. The robustness of the tree topology was evaluated with 1,000 bootstrap replicates. Bootstrap percentages greater than 50% are shown above branches. *Indigenous to Asia and Russia. **Indigenous to Asia. The sequences obtained in this study are marked by a black circle.

To obtain information about the long-term evolution of BLV in Asia, archived bat samples, which were collected in Asian countries were analyzed for the presence of endogenous deltaretrovirus sequences in their genome. Specifically, the DNA samples of 41 bat species from eight bat families found in 10 Asian countries were examined (Table 4). The endogenous deltaretrovirus *long terminal repeats* (*LTR*) were detected in 19 bat species. Among these bat species, 17 are indigenous and mainly distributed in Asia. Moreover, three species (*Rhinolophus pumilus*, *R. perditus*, and *Hipposideros turpis*) were endemic to the Ryukyu Islands, which located at the southern part of the Japanese archipelago. Since the Ryukyu Islands are surrounded by deeper marine straits and were not connected to the mainland during the whole Quaternary, these results indicated that endogenous deltaretrovirus sequences have circulated in Asia around the end of Neogene Period (Millien-Parra and Jaeger, 1999). Moreover, the ML phylogenetic analysis using partial *LTR* sequences obtained from the analyzed samples revealed that endogenous deltaretrovirus sequences obtained from bats endemic to Asia were closely related to the BLV *LTR* of lineage II (Figure 6). As some endogenous deltaretrovirus positive bat species were diverged and distributed in Asia during the late Miocene to Pliocene, these results indicate that BLV-related ancient deltaretroviruses circulated in Asia long before the emergence of BLV (Figure 6).

**TABLE 4:** List of the bat species analyzed.

Family	Scientific name	Sampling location		Sample IDs	LTR PCR positive samples/number of samples tested	Primer name in Supplementary Table 1 used for LTR amplification
		Country	Region			
**Rhinolophidae (*n* = 10)**						
	Rhinolophus nippon**	Japan	Honshu	OCUM7420	1/1	B
		South Korea	-	OCUM5272	0/1	
	Rhinolophus pumilus**	Japan	Ryukyu islands	OCUM6988	1/1	B
	Rhinolophus perditus**	Japan	Ryukyu islands	OCUM6038	1/1	B
	Rhinolophus monoceros**	Taiwan	-	OCUM6830	1/1	B
	Rhinolophus cornutus**	Japan	Honshu	OCUM6163	1/1	B
		China	Guangzhou	OCUM8059	1/1	B
	Rhinolophus macrotis**	China	Guangzhou	OCUM8079	1/1	B
	Rhinolophus formasae**	Taiwan	-	OCUM7972	1/1	B
	Rhinolophus rouxi**	China	Sichuan	OCUM7047	0/1	
**Hipposideridae (*n* = 9)**						
	Hipposideros turpis**	Japan	Ryukyu islands	OCUM6031, OCUM6036	2/2	A
	Hipposideros terasensis**	Taiwan	-	OCUM6847	0/1	
	Hipposideros bicolor**	China	Guangzhou	OCUM7790, OCUM7800	2/2	A
	Hipposideros armiger**	China	Guangzhou	OCUM7785	0/1	
	Hipposideros armiger**	Nepal	-	OCUM6256	0/1	
	Hipposideros larvatus**	Cambodia	-	OCUM8352	1/1	A
	Coelops frithi**	Taiwan	-	OCUM6834	1/1	A
**Pteropodidae (*n* = 2)**						
	Cynopterus sphinx**	Thailand	-	OCUM7872	0/1	
	Pteropus dasymallus**	Japan			1/1	B
**Miniopteridae (*n* = 2)**						
	Miniopterus fuliginosus**	Japan	Honshu	OCUM4814	1/1	A
	Miniopterus fuscus**	Japan	Ryukyu islands	OCUM7583	1/1	A
**Megadermatidae (*n* = 1)**						
	Megaderma lyra**	Thailand	-	OCUM7689	1/1	A
**Emballonuridae (*n* = 2)**						
	*Emballonura nivalis*	Borneo	-	OCUM889	0/1	
	Taphozous melanopogon**	Cambodia	-	OCUM8356	0/1	
**Vespertilionidae (*n* = 18)**						
	Tylonicteris pachypus**	China	Guangzhou	OCUM8100	0/1	
	*Scotophilus heathsii*	Tiwan	-	OCUM8101	0/1	
	Ia io**	China	Sichuan	OCUM7071	1/1	C
	Nyctalus aviator**	Japan	-	OCUM7225	0/1	
	Nyctalus furvus**	Japan	Hokkaido	RTMM309	0/1	
	Plecotus sacrimontis**	Japan	-	OCUM5366	0/1	
	Barbastella pacifica**	Japan	Hokkaido	HK01629*^a^*	0/1	
	Myotis gracilis*	Japan	Hokkaido	EZ1200*^a^*	0/1	
	Myotis petax*	Japan	Hokkaido	KK0021*^a^*	0/1	
	Myotis longicaudatus*	Japan	Hokkaido	KK0010*^a^*	1/1	C
	Myotis bombinus**	Japan	-	OCUM5545	0/1	
	Myotis pruinosus**	Japan	-	OCUM7495	1/1	C
	Myotis ikonnikovi*	Japan	Hokkaido	KK0062*^a^*	0/1	
	Murina ussuriensis*	Japan	Hokkaido	KK0063*^a^*	0/1	
	Murina hilgendorfi*	Japan	-	OCUM7413	0/1	
	Pipistrellus abramus*	Japan	Miyagi	KKT	0/1	
	Pipistrellus endoi*	Japan	Miyagi	KKT	0/1	
	Vespertilio sinensis*	Japan	Hokkaido	KK0203*^a^*	0/1	
**Molossidae (*n* = 1)**						
	Tadarida insignis*	Japan	Mie	KKT	0/1	

*Indigenous to Asia and Russia.

**Indigenous to Asia.

*-unspecified.*

*OCUM: Sample collection of the Osaka City University Graduate School of Medicine (collected by Dr. Masashi Harada). RTMM309: Sample belongs to the Rishiri Town Museum (provided by Dr. Kuniko Kawai). KKT: Samples provided by Dr. Kuniko Kawai.*

*^a^Samples belong to the Tokai University (collected by Dr. Kuniko Kawai).*

## Discussion

Bovine leukemia virus is highly prevalent in cattle worldwide, however, little is known about its emergence and evolutionary history. This in part reflects the fact that our current understanding of BLV diversity comes almost exclusively from domesticated, phenotypically and genetically selected taurine cattle. Native cattle are highly adapted to local environments and resources and are not so often exported to other countries compared to the major commercial cattle breeds (Ajmone-Marsan et al., 2010). Therefore, it was hypothesized that Asian native cattle could have harbored BLV, causing them to be important for virus emergence, maintenance, and spread (Chen et al., 2010; Qiu et al., 2012; Decker et al., 2014). This study represents the first analysis of genetic diversity of BLV in a wide range of native cattle population in Asia, and the first application of Bayesian phylogeographic analysis to gain new insights into the epidemiological and spatiotemporal dynamics of BLV. Together with the results from endogenous deltaretrovirus sequences obtained from the genome of indigenous Asian bats, these data provide evidence for origin and evolutionary dynamics of BLV.

The results of the phylodynamic and ancestral trait reconstruction analyses reveal that progenitor of BLV originally circulated in Asia (Figure 3B). This conclusion was also supported by the high RSPP value for Southeast Asia obtained from the BEAST analysis (RSPP = 0.227 for Southeast Asia; Figure 5B). Although our results are not consistent with the report of the first EBL cattle in Germany, Asia is still a potential geographic region where the emergence and maintenance of BLV could have occurred (Bendixen, 1963). BLV exhibits a relatively narrow host range and predominantly infects the *Bos* genus. Not only taurine cattle, but also diverse *Bos* species such as zebu, banteng, and gaur originated and were domesticated in Asia (Fuller, 2006; Melletti and Burton, 2014; Perez-Pardal et al., 2018). These species have been extensively cross-bred with other species of the *Bos* genus through the process of adaptation. This historical background may explain why the phylodynamic model inferred that Southeast Asia was the ancestral location of BLV origin. Further studies, including surveillance of diverse *Bos* species using a wide range of samples, will be needed to determine the more detailed geographic origin of BLV.

The results of ancestral trait reconstruction analysis reveal that zebu cattle were the source of BLV introduction into the taurine cattle population (Figure 3). This is also supported by the identification of a monophyletic clade, mainly constituted by zebu cattle and yak BLV (lineage II), sharing a MRCA with taurine BLV (lineage I). Lineage II also includes yak sequences, indicating that this animal should also be a source of cross-species transmission to taurine cattle. However, from the results of the Bayesian phylodynamic analysis and ancestral trait reconstruction analysis of lineage II, zebu cattle were reconstructed at the MRCA of the lineage II root in 100% (node ID: H and I in Figure 3A), suggesting that BLV in the yak population is the result of recent cross-species transmission from zebu cattle. These results are further supported by the positive selection analysis, which showed that the *BLVgp51* sequences from yak displayed a higher diversity and positive selection sites near predicted receptor binding sites (Figure 4). As the habitat of yak is limited to high altitude areas, it is concluded that the role of this animal played for the transmission cycle may be limited (Qiu et al., 2012).

The results of the Bayesian phylodynamic analysis and ancestral trait reconstruction analysis of lineage I indicated that the ancestral BLV sequence of Asian zebu cattle was introduced to taurine cattle not only in South America, but also in Europe (Figures 3B, 5, and route No. 31 of Table 3). Indeed, in the eighteenth century, zebu cattle were extensively exported from Asia to South America, mainly to Brazil, and were cross-bred with local taurine cattle for genetic improvement (Felius et al., 2014). Our results indicate that this process may have contributed to the spread of BLV in the taurine population. On the other hand, it remains unclear how the BLV ancestor reached European countries, because zebu cattle were not introduced in large numbers to European countries. A possible explanation of the BLV spread in Europe could be that in the eighteenth century, western European countries were directly involved in trade with Asian countries as well as with South America via the Atlantic Ocean. Their major transit ports were used during transit on the route from Asia to South America. As a result, large numbers of live cattle from diverse countries in Asia, South America, and Europe may have temporarily been held in the same port, or in quarantine facilities, causing the risk of increased virus transmission in Europe (Acemoglu et al., 2005). It is known that zebu cattle that were en route to Brazil from India introduced the rinderpest to Belgium via the port of Antwerp (Mammerickx, 2003). After the spread of BLV in Europe, genotype 4 spread to Russia, diversified to genotype 7, and was thus maintained, while it was eradicated in Europe (Polat et al., 2017).

The present analysis, however, identifies taurine cattle as the host responsible for the later dissemination of BLV worldwide over time. This probably began after World War II, and it coincided with a drastic increase of live cattle trade between continents (Ajmone-Marsan et al., 2010). The phylogenetic analysis suggests that the BLV genotype 1 is predominantly distributed worldwide as a pandemic genotype, and its source location is South America (Figure 3, MRCA: D) (Polat et al., 2017). The global dispersal of genotype 1 appears to have occurred in two steps, starting with the widespread export of the virus from South America via the United States to the rest of the world around 1950, followed by local diffusion within the countries where it was introduced (Figure 3). The initial step coincided with the worldwide distribution of the established commercial breeds to many other countries, and the second step coincided with the cross breeding with local populations (Polat et al., 2017). The phylogenetic analysis also demonstrates that the significant increase in genetic diversity observed during the late-1900s, coincided with the increase of international animal trade activities (Figure 5D; Ajmone-Marsan et al., 2010; Felius et al., 2014). This increase in genetic diversity, which occurred worldwide, may be explained by the adaptation of BLV to the local cattle that presented diverse genetic backgrounds (Figure 3). The increase was then followed by a notable decline, which was associated with the successful eradication programs adopted in European countries (Polat et al., 2017).

In this study, endogenous deltaretrovirus sequences, which were closely related to BLV lineage II, were detected in the bat genome of several indigenous Asian species (Table 4). Previous studies suggest that bat species were infected with the ancestors of endogenous deltaretroviruses around the end of the Paleogene and beginning of the Neogene (Hron et al., 2019). The inferred date corresponds closely with the adaptive radiation of several bat clades and species including endogenous deltaretrovirus positive bat species; for example, the tMRCA of the *Myotis* and the *Hipposideros* Asian clade is estimated to be 8.8 MYA and 19.9 MYA, and the time of migration and genetic isolation of the Japanese population of *Rhinolophus nippon* is estimated to be 0.5 MYA (Table 4; Ruedi et al., 2013; Amador et al., 2018; Ikeda and Motokawa, 2021). On the other hand, molecular dating estimates in previous studies have suggested that the tribe Bovini rapidly diversified into the three subtribes—Bovina, Bubalina, and Pseudorygina—during the late Middle Miocene (around 13 MYA) (Melletti and Burton, 2014). As these ancestors of bats and Bovini subtribes are currently more diversified in Asia, it can be assumed that ancient exogenous deltaretroviruses would circulate, endogenized to bat genome and would be maintained as they evolve at the host mutation rate of evolution. The *LTR* sequences of endogenous deltaretrovirus were also detected from bat endemic to Ryukyu islands. Ryukyu islands became isolated from mainland during the whole Quaternary (Millien-Parra and Jaeger, 1999; Yamada, 2017). It is possible that transmission of ancient deltaretrovirus occurred directly between animals of the tribe Bovini and bat species or between hosts via an intermediate host, or were transmitted to both hosts from unknown host. Regardless of the route of transmission, having the result of our study as a presumption that ancient exogenous deltaretrovirus were circulated in Asia before the isolation of Ryukyu islands, we can assume that BLV-related ancient deltaretroviruses in the past would circulate in Asia long before the emergence of this virus. Interestingly, while human T-lymphotropic virus (HTLV) was inferred to have emerged as a result of cross-species transmission of simian T-lymphotropic virus (STLV) from animal reservoirs in Africa, and several previous studies pointed out that STLV originated in Asia. Therefore, these observations suggest that this region is likely to be the center of origin of deltaretroviruses (Slattery et al., 1999; Reid et al., 2016; Afonso et al., 2019). Further analysis is needed to detect other natural hosts of BLV, because the infection rate in zebu cattle is relatively low for the maintenance of deltaretrovirus as a reservoir (Mwenda et al., 1999; Takemura et al., 2002; Traina-Dorge et al., 2005; Jegado et al., 2019).

This study has several limitations, as the phylodynamic models were constructed using *BLVgp51* sequences, and the evolutionary history of other gene sequences was not included. Moreover, the host species of most of the analyzed sequences collected before 2000 were represented by taurine cattle. Although the topologies of ML phylogenetic tree of four BLV open reading frames were mostly compatible with each other, and it was shown to be appropriate for the trait analyses conducted in our previous study, the analysis of full length genomes from various species collected on different years could also provide basic information to deepen the long history of viral evolution (Ohnuki et al., 2021).

In summary, by analyzing BLV sequences from Asian native cattle and endogenous deltaretrovirus sequences from indigenous Asian bat species, it was inferred that ancient BLV emerged and circulated in Asia long before it was detected in Europe in the late 1800s. The potential cross-species transmission of BLV to taurine cattle, possibly through Asian zebu cattle, is likely to have occurred around the late 1800s in South America and Europe. These results can not only elucidate the mechanisms of BLV dissemination worldwide, but also provide new insights into the evolution of deltaretroviruses.

## Materials and Methods

### Bovine Sample Collection

Blood samples were collected from 256 zebu cattle in six Asian countries. Each sample was unrelated and taken from one head per farm. The detailed sampling method has been described in our previous work (Lwin et al., 2018b). In addition, samples from 37 zebu cattle in Madagascar were also collected, because this population is closely related to Asian zebu populations (Table 1; Kaufman, 2008). Blood samples were also collected from 16 taurine cattle (*Bos taurus*) in the Republic of Kazakhstan. Blood samples and skin tissue samples from 268 yaks (*Bos grunniens*) and 16 water buffaloes (*Bubalus bubalis*) were collected in four countries (Kyrgyzstan, China, Pakistan, and Nepal) (Guo et al., 2006; Qiu et al., 2012). The DNA extraction procedure and the information on samples have been included in our previous study (Yonesaka et al., 2016; Lwin et al., 2018a,b).

### Detection of Bovine Leukemia Virus Proviral DNA and Sequencing Analysis

A 1304 bp fragment of the complete *BLVgp51* gene was amplified by PCR using KOD FX Neo (TOYOBO, Osaka, Japan) with following primers (Forward Primer 5′-AGA TGG GAG CTA CAC CAT TCA-3′, Reverse Primer 5′-CAC AGA GGC CAC ATT AAG A-3′) to detect BLV proviral sequences in the genome of zebu cattle, taurine cattle and water buffaloes. The following cycling conditions were used: 94°C for 2 min, followed by 40 cycles of denaturation at 98°C for 10 s, and extension at 68°C for 60 s. The amplified PCR fragments were purified by QIAquick gel extraction kit (QIAGEN, Hiden, Germany), and were then sequenced (FASMAC, Atsugi, Japan). The sequences included a 903 bp sequence coding the complete BLV envelope GP51 region, corresponding to nucleotide positions 4826–5738 on the pBLV903 genome (GenBank accession number EF600696). The obtained sequences were analyzed in Sequencher™5.2.4 (GeneCodes, Michigan, United States).

A 198 bp fragment of the LTR of the BLV proviral genome in yak was amplified by nested PCR using PrimeSTAR GXL DNA polymerase (Takara, Shiga, Japan) with the following primers: BLTR256F (5′-GAG CTC TCT TGC CCG AGA C-3′) and BLTR453R (5′-GAA ACA AAC GCG GGT GCA AGC CAG-3′) for first PCR, and BLTR306F (5′-GTA AGG CAA ACC ACG GTT T-3′) and BLTR408R (5′-AGG AGG CAA AGG AGA GAG T-3′) for second PCR, as previously described (Polat et al., 2015). The following cycling conditions were used: 45 cycles of denaturation at 98°C for 10 s, annealing at 60°C for 15 s, extension at 68°C for 30 s.

### The Maximum Likelihood Phylogenetic Tree

A total of 699 *BLVgp51* partial sequences (444 bp) as well as collection date and location data were retrieved from GenBank. These sequences were aligned with 13 sequences obtained in this study using MEGA7 software (Kumar et al., 2016). The best-fitting nucleotide substitution model was selected using the Akaike information criterion corrected (AICc). A ML phylogenetic tree of the *BLVgp51* sequences was inferred by applying the Kimura 2-parameter model plus gamma distribution (K2+G) model. The reliability of the phylogenetic relationships was evaluated by non-parametric bootstrap analysis with 1,000 replicates.

### Phylogenetic Reconstruction and Phylodynamic Analysis of Bovine Leukemia Virus

To analyze the evolutionary history of BLV, the time to the most recent common ancestor (tMRCA) and the effective population size were estimated by employing the Bayesian MCMC approach in BEAST v 2.4.8 (Bouckaert et al., 2014). Coalescent theory has been applied to phylogenetic methods to reconstructing the epidemic history of viruses, providing estimates of origins, dating of common ancestors, and population dynamics. The best nucleotide substitution model for the Bayesian MCMC-derived phylogenetic tree was selected based on the Bayesian information criterion (BIC) values in MEGA7 (Kumar et al., 2016). The Hasegawa-Kishino-Yano (HKY) model that showed the lowest BIC value was used for this analysis. The temporal scale of the evolutionary process was inferred from the sampling dates of the sequences using relaxed clock models (exponential and lognormal relaxed clocks) and four demographic models (two parametric priors, coalescent constant population and coalescent exponential population; two non-parametric priors, coalescent extended skyline and coalescent Bayesian skyline). Therefore, the best fitting demographic models were selected based on the BF test and Akaike’s information criterion for MCMC (AICM) values using Tracer v1.5 (Rambaut and Drummond, 2007). The BF and AICM analyses also showed that the exponential relaxed clock was better than the lognormal one in fitting the data. Also, under the exponential relaxed clock, the BF and AICM analyses showed that the coalescent exponential population fitted the data better than other demographic models did. For the Bayesian MCMC analysis, HKY+exponential relaxed clock+coalescent exponential population were selected. A total of 156 *BLVgp51* sequences (sequence length, 502–903 bp) were used for the analysis in BEAST. First, the sequences with collection date, country, and host information were obtained from the database. A 75% random sampling was performed for countries with more than 10 sequences registered, because the BLV database was biased toward many closely related sequences in the same country. Then, Bayesian MCMC simulations for 400 million cycles, sampling every 10,000 states, and 10% burn-in were applied to infer the BLV evolutionary phylogenetic tree under the best fitting model. The convergence was assessed by estimating the effective sample size (ESS) (>200) of a parameter sampled from the Bayesian MCMC using Tracer v 1.5 (Rambaut and Drummond, 2007). The maximum clade credibility (MCC) tree was constructed using TreeAnnotator v1.8.0 (Rambaut and Drummond, 2016), and finally, the MCC tree was generated in FigTree v 1.4.4 (Rambaut, 2019).

### Ancestral State Reconstruction

To study the evolutionary history of BLV, ancestral state reconstruction analyses were performed using Mesquite v 3.61 (Maddison, 2016). The *BLVgp51* MCC tree previously estimated in this study (Figure 2) was used for the input data. The ancestral states of each BLV in relation to its host and geography were reconstructed using a parsimony approach. The minimum number of trait changes along the tree that are necessary to explain the present host state and sampling location associations at the tree tips were calculated to estimate the evolutionary process.

### Positive Selection Analysis

Of the 156 *BLVgp51* sequences (903 bp) used in this study, 63 were classified as lineage II, and were used to test the positive selection. The phylogenetic trees were reconstructed by employing the Bayesian MCMC method in BEAST v 2.4.8, and the resulting trees were then used for detecting positive selection. For the detection of positive selection pressure, the analysis was performed with the PAML 4.8 site model. Amino acid sites under positive selection pressure were identified by posterior probabilities through the Bayes empirical Bayes (BEB) method (Yang, 2007). The selected sites were compared to those obtained from the previous study of predicted BLV receptor binding sites (Corredor et al., 2018).

### Spatial Phylogenetic Reconstruction of Evolutionary Dynamics

The global geographic origins of BLV and its significant dispersal routes between infected countries were inferred using discrete-state ancestral reconstruction methods in BEAST v 2.4.8. Each BLV isolate was assigned an individual location state, based on the country/region of the sample location data. Diffusions among the location states were modeled using an asymmetric model, with Bayesian stochastic search variable selection. The same settings as those described for the Bayesian phylogenetic dating analyses above were used. The resulting diffusion rates were then used to calculate BF in SPREAD (Bielejec et al., 2011). The migration pathways were interpreted as “supported” when the BF was ≥ 3. To minimize the impact of sampling bias, countries with a sample size of 10 or more were kept below 20% of the sample.

### Detection of Bat Endogenous Deltaretrovirus and Its Relationship to Bovine Leukemia Virus

To assess the presence of endogenous deltaretrovirus sequences, DNA or tissue samples obtained from 46 bats originated in Asia—comprising 41 species and eight families were analyzed. The DNA was extracted by phenol chloroform method. The *LTR* and *gag* regions of endogenous deltaretroviruses were amplified by PCR using PrimeSTAR GXL DNA polymerase (Takara, Shiga, Japan) (information on the specific primers used is included in Supplementary Table 1). The following cycling conditions were used: 40 cycles of denaturation at 94°C for 10 s, annealing at 60°C for 15 s, and extension at 68°C for 45 s. The amplified fragments were purified by QIAquick gel extraction kit (QIAGEN, Hiden, Germany), and were then sequenced (FASMAC, Atsugi, Japan). All amplicons were sequenced directly, and sequences with ambiguous positions excluded from further analysis. To determine the evolutionary relationships of the obtained sequences among endogenous and exogenous deltaretroviruses, the *LTR* sequences were aligned using ClustalW in MEGA7, and a phylogenetic tree was estimated using the ML method with the K2+G model of nucleotide substitution. To evaluate the robustness of each node, bootstrap resampling analysis was performed with 1,000 replicates.

## Data Availability Statement

The datasets presented in this study can be found in online repositories. The names of the repository/repositories and accession number(s) can be found in the article/Supplementary Material.

## Ethics Statement

The animal study was reviewed and approved by Kobe University Animal Experimentation Regulations and the Animal Research Committee at Tokyo University of Agriculture, and we confirm that all experiments were performed in accordance with the committee’s guidelines and regulations. Written informed consent was obtained from the owners for the participation of their animals in this study.

## Author Contributions

TK and KN conceived and designed the experiments and wrote the manuscript. KN performed the wet lab experiments. KN, TY, and TK performed the phylogenetic data analyses. KN, TY, MN, FK, SS, KK, HM, MH, and JL contributed to the yak, water buffalo, bats, and native cattle samples. TY, MN, YT, KK, JL, HM, and TK coordinated the project. TY, MN, KI, KK, JL, and HM provided guidance and expertised on the history of native cattle, yak, bat, and endogenous retroviruses. All authors read and approved the final manuscript.

## Conflict of Interest

The authors declare that the research was conducted in the absence of any commercial or financial relationships that could be construed as a potential conflict of interest.

## Publisher’s Note

All claims expressed in this article are solely those of the authors and do not necessarily represent those of their affiliated organizations, or those of the publisher, the editors and the reviewers. Any product that may be evaluated in this article, or claim that may be made by its manufacturer, is not guaranteed or endorsed by the publisher.

## References

[B1] Acemoglu D. Johnson S. Robinson J. (2005). The rise of Europe: atlantic trade, institutional change, and economic growth. *Am. Econ. Rev.* 95 546–579.

[B2] Afonso P. V. Cassar O. Gessain A. (2019). Molecular epidemiology, genetic variability and evolution of HTLV-1 with special emphasis on African genotypes. *Retrovirology* 16 1–15. 10.1186/s12977-019-0504-z 31842895 PMC6916231

[B3] Aida Y. Murakami H. Takahashi M. Takeshima S. N. (2013). Mechanisms of pathogenesis induced by bovine leukemia virus as a model for human T-cell leukemia virus. *Front. Microbiol.* 4:328. 10.3389/fmicb.2013.00328 PMC3820957 24265629

[B4] Ajmone-Marsan P. Garcia J. F. Lenstra J. A. (2010). On the origin of cattle: how aurochs became cattle and colonized the world. *Evol. Anthropol.* 19 148–157.

[B5] Amador L. I. Arévalo R. L. M. Almeida F. C. Catalano S. A. Giannini N. P. (2018). Bat systematics in the light of unconstrained analyses of a comprehensive molecular supermatrix. *J. Mammal. Evol.* 25 37–70.

[B6] Bendixen H. J. (1963). Preventive Measures in Cattle Leukemia: leukosis Enzootica Bovis. *Ann. N. Y. Acad. Sci.* 108 1241–1267. 10.1111/j.1749-6632.1963.tb13448.x 14081503

[B7] Benitez O. J. Norby B. Bartlett P. C. Maeroff J. E. Grooms D. L. (2020). Impact of bovine leukemia virus infection on beef cow longevity. *Prev. Vet. Med.* 181:105055. 10.1016/j.prevetmed.2020.105055 32593082

[B8] Bielejec F. Rambaut A. Suchard M. A. Lemey P. (2011). SPREAD: spatial phylogenetic reconstruction of evolutionary dynamics. *Bioinformatics*. 15, 2910–2912. 10.1093/bioinformatics/btr481 21911333 PMC3187652

[B9] Boeke J. D. Stoye J. P. (1997). “Retrotransposons, Endogenous Retroviruses, and the Evolution of Retroelements,” in *Retroviruses*, eds Coffin J. M. Hughes S. H. Varmus H. E. (New York, NY: Cold Spring Harbor). 21433351

[B10] Bouckaert R. Heled J. Kuhnert D. Vaughan T. Wu C. H. Xie D. (2014). BEAST 2: a software platform for Bayesian evolutionary analysis. *PLoS Comput. Biol.* 10:e1003537. 10.1371/journal.pcbi.1003537 PMC3985171 24722319

[B11] Chen S. Lin B. Z. Baig M. Mitra B. Lopes R. J. Santos A. M. (2010). Zebu cattle are an exclusive legacy of the South Asia neolithic. *Mol. Biol. Evol.* 27 1–6. 10.1093/molbev/msp213 19770222

[B12] Constable P. D. Hinchcliff K. W. Done S. H. Gruenberg W. Radostits O. M. (2017). *Veterinary medicine : a textbook of the diseases of cattle, horses, sheep, pigs and goats.* Amsterdam: Elsevier.

[B13] Corredor A. P. González J. Baquero L. A. Curtidor H. Olaya-Galán N. N. Patarroyo M. A. (2018). In silico and in vitro analysis of boAP3d1 protein interaction with bovine leukaemia virus gp51. *PLoS One* 13:e0199397. 10.1371/journal.pone.0199397 29928016 PMC6013181

[B14] Decker J. E. Mckay S. D. Rolf M. M. Kim J. Molina Alcala A. Sonstegard T. S. (2014). Worldwide patterns of ancestry, divergence, and admixture in domesticated cattle. *PLoS Genet.* 10:e1004254. 10.1371/journal.pgen.1004254 24675901 PMC3967955

[B15] Farkasova H. Hron T. Paces J. Hulva P. Benda P. Gifford R. J. (2017). Discovery of an endogenous Deltaretrovirus in the genome of long-fingered bats (Chiroptera: Miniopteridae). *Proc. Natl. Acad. Sci. U S A* 114 3145–3150. 10.1073/pnas.1621224114 28280099 PMC5373376

[B16] Felius M. Beerling M.-L. Buchanan D. S. Theunissen B. Koolmees P. A. Lenstra J. A. (2014). On the History of Cattle Genetic Resources. *Diversity* 6 705–750.

[B17] Fuller D. Q. (2006). Agricultural origins and frontiers in South Asia: a working synthesis. *J. World Prehist.* 20 1–86.

[B18] Guo S. Savolainen P. Su J. Zhang Q. Qi D. Zhou J. (2006). Origin of mitochondrial DNA diversity of domestic yaks. *BMC Evol. Biol.* 6:73. 10.1186/1471-2148-6-73 16995938 PMC1626082

[B19] Hayward J. A. Tachedjian M. Kohl C. Johnson A. Dearnley M. Jesaveluk B. (2020). Infectious KoRV-related retroviruses circulating in Australian bats. *Proc. Natl. Acad. Sci. U S A* 117 9529–9536. 10.1073/pnas.1915400117 32284399 PMC7196810

[B20] Hron T. Elleder D. Gifford R. J. (2019). Deltaretroviruses have circulated since at least the Paleogene and infected a broad range of mammalian species. *Retrovirology* 16:33. 10.1186/s12977-019-0495-9 31775783 PMC6882180

[B21] Ikeda Y. Motokawa M. (2021). Phylogeography of the Japanese greater horseshoe bat Rhinolophus nippon (Mammalia: Chiroptera) in Northeast Asia: new insight into the monophyly of the Japanese populations. *Ecol. Evol.* 11 18181–18195. 10.1002/ece3.8414 35003666 PMC8717313

[B22] Irving A. T. Ahn M. Goh G. Anderson D. E. Wang L. F. (2021). Lessons from the host defences of bats, a unique viral reservoir. *Nature* 589 363–370. 10.1038/s41586-020-03128-0 33473223

[B23] Jegado B. Kashanchi F. Dutartre H. Mahieux R. (2019). STLV-1 as a model for studying HTLV-1 infection. *Retrovirology* 16:41. 10.1186/s12977-019-0503-0 31843020 PMC6915939

[B24] Kaufman J. C. (2008). *Greening the great red island : Madagascar in nature and culture. Africa Institute Occasional Paper.* Pretoria: Africa Institute of South Africa.

[B25] Kumar S. Stecher G. Tamura K. (2016). MEGA7: molecular Evolutionary Genetics Analysis Version 7.0 for Bigger Datasets. *Mol. Biol. Evol.* 33 1870–1874. 10.1093/molbev/msw054 27004904 PMC8210823

[B26] Lwin M. Mon S. L. Y. Yamanaka H. Nagano Y. Mannen H. Faruque M. O. (2018b). Genetic diversities and population structures of four popular Myanmar local cattle breeds. *Anim. Sci. J.* 89 1648–1655. 10.1111/asj.13112 30318818

[B27] Lwin M. Mon S. L. Y. Nagano Y. Kawabe K. Mannen H. Okamoto S. (2018a). Genetic diversity of Myanmar cattle breeds using complete mitochondrial D-loop sequence. *J. Anim. Genet.* 46 57–67.

[B28] Ma J. G. Zheng W. B. Zhou D. H. Qin S. Y. Yin M. Y. Zhu X. Q. (2016). First Report of Bovine Leukemia Virus Infection in Yaks (*Bos mutus*) in China. *Biomed. Res. Int.* 2016:9170167. 10.1155/2016/9170167 27340671 PMC4909904

[B29] Maddison W. P. A. D. R. M. (2016). *Measquite: a modular system for evolutionary analysis. Version 3.61.*

[B30] Mammerickx M. (2003). La peste bovine, Jules Bordet et le centre Sérumigène de Cureghem. *Ann. Med. Vét.* 147 197–205.

[B31] Melletti M. Burton J. (2014). *Ecology, Evolution and Behaviour of Wild Cattle: Implications for Conservation.* Cambridge, MA: Cambridge University Press.

[B32] Millien-Parra V. Jaeger J. J. (1999). Island biogeography of the Japanese terrestrial mammal assemblages: an example of a relict fauna. *J. Biogeogr.* 26 959–972.

[B33] Mwenda J. M. Sichangi M. W. Isahakia M. Van Rensburg E. J. Langat D. K. (1999). The prevalence of antibodies to simian T-cell leukaemia/lymphotropic virus (STLV-I) in non-human primate colonies in Kenya. *Ann. Trop. Med. Parasitol.* 93 289–297. 10.1080/00034989958555 10562831

[B34] Ohnuki N. Kobayashi T. Matsuo M. Nishikaku K. Kusama K. Torii Y. (2021). A target enrichment high throughput sequencing system for characterization of BLV whole genome sequence, integration sites, clonality and host SNP. *Sci. Rep.* 11:4521. 10.1038/s41598-021-83909-3 33633166 PMC7907107

[B35] OIE (2021). *Manual of Diagnostic Tests and Vaccines for Terrestrial Animals.* Paris: OIE.

[B36] Ott S. L. Johnson R. Wells S. J. (2003). Association between bovine-leukosis virus seroprevalence and herd-level productivity on US dairy farms. *Prev. Vet. Med.* 61 249–262. 10.1016/j.prevetmed.2003.08.003 14623410

[B37] Perez-Pardal L. Sanchez-Gracia A. Alvarez I. Traore A. Ferraz J. B. S. Fernandez I. (2018). Legacies of domestication, trade and herder mobility shape extant male zebu cattle diversity in South Asia and Africa. *Sci. Rep.* 8:18027. 10.1038/s41598-018-36444-7 30575786 PMC6303292

[B38] Perry B. D. Grace D. Sones K. (2013). Current drivers and future directions of global livestock disease dynamics. *Proc. Natl. Acad. Sci. U S A* 110 20871–20877. 10.1073/pnas.1012953108 21576468 PMC3876220

[B39] Polat M. Ohno A. Takeshima S. N. Kim J. Kikuya M. Matsumoto Y. (2015). Detection and molecular characterization of bovine leukemia virus in Philippine cattle. *Arch. Virol.* 160 285–296. 10.1007/s00705-014-2280-3 25399399

[B40] Polat M. Takeshima S. N. Aida Y. (2017). Epidemiology and genetic diversity of bovine leukemia virus. *Virol. J.* 14:209. 10.1186/s12985-017-0876-4 29096657 PMC5669023

[B41] Qiu Q. Zhang G. Ma T. Qian W. Wang J. Ye Z. (2012). The yak genome and adaptation to life at high altitude. *Nat. Genet.* 44 946–949. 10.1038/ng.2343 22751099

[B42] Rambaut A. (2019). *Figtree v 1.4.4.*

[B43] Rambaut A. Drummond A. (2007). *Tracer v1.5. beast. bio. ed. ac. uk/Tracer.*

[B44] Rambaut A. Drummond A. (2016). *TreeAnnotator version 1.8.0.*

[B45] Reid M. J. Switzer W. M. Schillaci M. A. Ragonnet-Cronin M. Joanisse I. Caminiti K. (2016). Detailed phylogenetic analysis of primate T-lymphotropic virus type 1 (PTLV-1) sequences from orangutans (Pongo pygmaeus) reveals new insights into the evolutionary history of PTLV-1 in Asia. *Infect Genet. Evol.* 43 434–450. 10.1016/j.meegid.2016.05.036 27245152 PMC11332081

[B46] Roberts T. E. (2006). History, ocean channels, and distance determine phylogeographic patterns in three widespread Philippine fruit bats (Pteropodidae). *Mol. Ecol.* 15 2183–2199. 10.1111/j.1365-294X.2006.02928.x 16780434

[B47] Ruedi M. Stadelmann B. Gager Y. Douzery E. J. Francis C. M. Lin L. K. (2013). Molecular phylogenetic reconstructions identify East Asia as the cradle for the evolution of the cosmopolitan genus Myotis (Mammalia, Chiroptera). *Mol. Phylogenet. Evol.* 69 437–449. 10.1016/j.ympev.2013.08.011 23988307

[B48] Scheu A. Powell A. Bollongino R. Vigne J. D. Tresset A. Cakirlar C. (2015). The genetic prehistory of domesticated cattle from their origin to the spread across Europe. *BMC Genet.* 16:54. 10.1186/s12863-015-0203-2 26018295 PMC4445560

[B49] Sihvonen L. (2015). Enzootic bovine leukosis. *EFSA J.* 13:63. 10.2903/j.efsa.2015.4188 PMC13151565 42109774

[B50] Slattery J. P. Franchini G. Gessain A. (1999). Genomic evolution, patterns of global dissemination, and interspecies transmission of human and simian T-cell leukemia/lymphotropic viruses. *Genome Res.* 9 525–540. 10400920

[B51] Takemura T. Yamashita M. Shimada M. K. Ohkura S. Shotake T. Ikeda M. (2002). High prevalence of simian T-lymphotropic virus type L in wild ethiopian baboons. *J. Virol.* 76 1642–1648. 10.1128/jvi.76.4.1642-1648.2002 11799159 PMC135919

[B52] Traina-Dorge V. L. Lorino R. Gormus B. J. Metzger M. Telfer P. Richardson D. (2005). Molecular epidemiology of simian T-cell lymphotropic virus type 1 in wild and captive sooty mangabeys. *J. Virol.* 79 2541–2548. 10.1128/JVI.79.4.2541-2548.2005 15681454 PMC546543

[B53] Wang M. Wang Y. Baloch A. R. Pan Y. Xu F. Tian L. (2018). Molecular epidemiology and characterization of bovine leukemia virus in domestic yaks (Bos grunniens) on tshe Qinghai-Tibet Plateau, China. *Arch. Virol.* 163 659–670. 10.1007/s00705-017-3658-9 29224130

[B54] Yamada F. (2017). The birth of two new national parks on the Central Ryukyus in the Ryukyu Chain and the task of a World Natural Heritage Site. *Mammal. Sci.* 57 183–194.

[B55] Yang Z. (2007). PAML 4: phylogenetic analysis by maximum likelihood. *Mol. Biol. Evol.* 24 1586–1591. 17483113 10.1093/molbev/msm088

[B56] Yonesaka R. Sasazaki S. Yasue H. Niwata S. Inayoshi Y. Mukai F. (2016). Genetic structure and relationships of 16 Asian and European cattle populations using DigiTag2 assay. *Anim. Sci. J.* 87 190–196. 10.1111/asj.12416 26260416 PMC5042107

